# Higher Adenoma Detection Rates at Screening Associated With Lower Long-Term Colorectal Cancer Incidence and Mortality

**DOI:** 10.1016/j.cgh.2020.09.020

**Published:** 2022-02

**Authors:** Amanda J. Cross, Emma C. Robbins, Brian P. Saunders, Stephen W. Duffy, Kate Wooldrage

**Affiliations:** ∗ Cancer Screening and Prevention Research Group, Department of Surgery and Cancer, Imperial College London, London, United Kingdom; ‡ Wolfson Unit for Endoscopy, St Mark's Hospital, London, United Kingdom; § Centre for Cancer Prevention, Wolfson Institute of Preventive Medicine, Queen Mary University, London, United Kingdom

**Keywords:** Adenoma Detection Rate, Flexible Sigmoidoscopy, Colorectal Cancer, Screening, ADR, adenoma detection rate, BCSP, bowel cancer screening program, CRC, colorectal cancer, FS, flexible sigmoidoscopy, HR, hazard ratio, NNS, number needed to screen, RR, rate ratio, UKFSST, the UK Flexible Sigmoidoscopy Screening Trial

## Abstract

**Background & Aims:**

Detection and removal of adenomas reduces colorectal cancer (CRC) risk. The impact of adenoma detection rates (ADRs) on long-term CRC incidence and mortality is unknown. We investigated this using data from the UK Flexible Sigmoidoscopy Screening Trial.

**Methods:**

Of 167,882 UK Flexible Sigmoidoscopy Screening Trial participants, 40,085 were in the intervention arm and underwent flexible sigmoidoscopy screening at 13 trial centers. The median follow-up time was 17 years. At each center, 1 endoscopist performed most flexible sigmoidoscopies. Multivariable logistic regression was used to classify centers into high-, intermediate-, and low-detector groups based on their main endoscopist’s ADR. We calculated the incidence and mortality of distal and all-site CRC, and estimated hazard ratios (HRs) with 95% CIs using Cox regression.

**Results:**

Five, 4, and 4 centers, respectively, were classified into the high-detector, intermediate-detector, and low-detector groups. The average ADRs in each respective group were 15%, 12%, and 9%. Distal CRC incidence and mortality were reduced among those screened compared with controls in all groups, and effects of screening varied significantly by detector ranking, with larger reductions in incidence and mortality seen in the high-detector group (incidence: HR, 0.34; 95% CI, 0.27–0.42; mortality: HR, 0.22, 95% CI, 0.13–0.37) than in the low-detector group (incidence: HR, 0.55; 95% CI, 0.44–0.68; mortality: HR, 0.54; 95% CI, 0.34–0.86). Similar results were observed for all-site CRC, with larger effects seen in the high-detector (incidence: HR, 0.58; 95% CI, 0.50–0.67; mortality: HR, 0.52; 95% CI, 0.39–0.69) than in the low-detector group (incidence: HR, 0.72; 95% CI, 0.61–0.85; mortality: HR, 0.68; 95% CI, 0.51–0.92), although the heterogeneity was not statistically significant.

**Conclusions:**

Higher ADRs at screening provide greater long-term protection against CRC incidence and mortality. Isrctn.org, number: ISRCTN28352761.

See editorial on page e25.

**What You Need to Know**

**Background**

Adenoma detection rates (ADRs) are associated inversely with risks of colorectal cancer (CRC) occurring within a few years after a negative endoscopy, but the impact of ADRs on long-term CRC outcomes is unknown.

**Findings**

Higher ADRs in the UK Flexible Sigmoidoscopy Screening Trial resulted in greater long-term protection against CRC incidence and mortality. Higher ADRs largely reflected improved detection of small (<10 mm) adenomas.

**Implications for patient care**

The ADR is a vitally important quality indicator for endoscopy, and widespread ADR improvement is needed to maximize the public health benefits of endoscopic screening.

Detection and removal of adenomas is the cornerstone of colorectal cancer (CRC) screening.^1^ Flexible sigmoidoscopy (FS), which examines the sigmoid colon and rectum, is used in several CRC screening programs and is highly effective at reducing CRC incidence and mortality.^2^, ^3^, ^4^ In the UK Flexible Sigmoidoscopy Screening Trial (UKFSST), a FS screen between ages 55 and 64 years reduced CRC incidence and mortality by 35% and 41%, respectively, after 17 years.^3^ These results led to the introduction of FS screening at age 55 years in the English Bowel Cancer Screening Program (BCSP).^5^

The one-off nature of FS screening means it is important to maximize the quality and efficacy of the procedure. The adenoma detection rate (ADR) is a key endoscopy quality indicator.^6^ In the UKFSST, ADRs varied widely and this reflected differences in endoscopist performance.^7^ It is intuitive to hypothesize that the more adenomas detected and removed at screening, the greater the protection offered against CRC. Studies have shown that higher ADRs at screening FS and colonoscopy are associated with lower risks of CRC being diagnosed within a few years (typically 3–5 years) after a negative screen.^8^, ^9^, ^10^, ^11^, ^12^ However, it has not been shown whether this translates into lower CRC incidence and mortality in the long-term.

The UKFSST, comprising 167,882 participants of whom 40,085 underwent FS screening by endoscopists with variable ADRs, provided an opportunity to examine associations between ADRs and long-term CRC incidence and mortality.^3^

## Methods

### Study Design and Participants

**Supplementary Figure 1:**
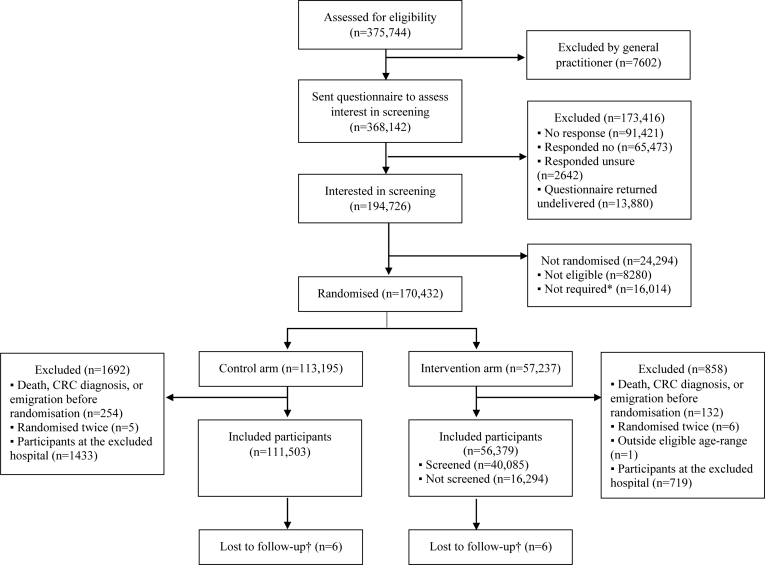
Trial profile flow diagram. ∗A total of 16,014 individuals were not required because the attendance of 40,000 participants at screening for which the trial was funded had been reached. †In total, 12 participants were lost to follow-up because they could not be traced through national data sources; 6 from the control arm and 6 from the intervention arm, of whom 2 had undergone screening. CRC, colorectal cancer.

The UKFSST recruited participants from general practices serving 14 UK hospitals from 1994 to 1999.^2^^,^^3^^,^^7^^,^^13^ Men and women aged 55 to 64 years were eligible unless they had a history of CRC, adenomas, or inflammatory bowel disease; a life expectancy of fewer than 5 years; a sigmoidoscopy or colonoscopy within the previous 3 years; or could not provide informed consent. Eligible individuals were randomized in a parallel design (1:2) to the intervention arm (n = 57,237), who were offered a FS screen, or the control arm (n = 113,195), who were not contacted (Supplementary Figure 1).^2^^,^^3^^,^^7^^,^^13^

At each hospital, 1 endoscopist performed nearly all FS examinations. All endoscopists used the same endoscope model and examination protocols and knew they would be observed.^13^

Endoscopists were encouraged to remove every polyp smaller than 10 mm, apart from those smaller than 3 mm in the distal rectum and considered to be benign hyperplastic polyps. Participants with more than 3 adenomas, an adenoma 10 mm or larger, with villous/tubulovillous histology, or high-grade dysplasia, 20 or more hyperplastic polyps above the rectum, or malignancy were offered a follow-up colonoscopy and then surveillance, typically involving 2 or more surveillance colonoscopies performed at 3-year intervals. Participants without these findings were not referred for colonoscopy follow-up evaluation or surveillance.

Cancer registrations, death certificate data, and emigration data were obtained from cancer registries, the National Health Service (NHS) Central Register, NHS Digital, National Services Scotland, and the Office for National Statistics. CRCs were defined by the International Classification of Diseases, 10th revision, as follows: proximal cancer was defined as C18.0 to C18.6 (descending colon to cecum); and distal cancer was defined as C18.7, C19, and C20 (rectum and sigmoid colon). CRC morphologies were defined by International Classification of Diseases for Oncology, 2nd edition, including codes relating to invasive adenocarcinomas and carcinoma not otherwise specified for cancers without diagnostic pathology.^3^

### Statistical Analysis

This analysis included follow-up data through 2014. Primary outcomes were distal CRC incidence and mortality. Secondary outcomes were all-site CRC incidence and mortality and the number needed to screen (NNS) to prevent 1 all-site CRC diagnosis or death. We examined distal CRC as our primary outcome because the UKFSST screening protocol had a negligible impact on proximal cancers.^2^^,^^3^

We excluded 1 UKFSST center because it had fewer participants (n = 2152) than the other centers and there were 2 main endoscopists rather than 1. The additional exclusion of 386 individuals owing to events preceding randomization (death, CRC diagnosis, or emigration), 11 who were randomized twice, and 1 outside of the eligible age range left 56,379 and 111,503 participants in the intervention and control arms, respectively, of whom 12 were lost to follow-up (Supplementary Figure 1).

We estimated ADRs for each center, calculated as the proportion of participants screened by the main endoscopist at that center who had 1 or more adenomas detected during screening. We considered any distal adenomas found at follow-up colonoscopy in this calculation because the UKFSST protocol stipulated that any polyp or adenoma 10 mm or larger detected at FS should be left intact for removal at colonoscopy. We used the same method to calculate detection rates of small (<10 mm), large (≥10 mm), and advanced (≥10 mm, villous/tubulovillous histology, or high-grade dysplasia) adenomas and polyps. We estimated average numbers of adenomas and polyps detected per 100 screened participants.

We assessed correlations between ADRs and detection rates of small adenomas/polyps, large adenomas/polyps, and advanced adenomas using the Spearman rank correlation coefficient.

Centers were ranked in descending order by ADRs. Multivariable logistic regression was used to classify centers into high-, intermediate-, and low-detector ranking groups, including ADR ranking and the sex and age of participants as explanatory variables.^7^

We estimated CRC incidence and mortality for each detector group. For distal CRC incidence, we included the earliest diagnosed distal CRC, even if proximal cancer had been diagnosed previously. For all-site CRC incidence, we counted the earliest diagnosed CRC only. Deaths in participants with distal and proximal cancers (n = 17) were not included as distal CRC-related because we could not determine which cancer caused death. We censored time-to-event data at emigration, death, or end of follow-up. Kaplan–Meier analyses showed time to CRC diagnosis or death.

Intention-to-treat analyses compared CRC incidence and mortality among the invited to screening and control arms, using Cox proportional-hazards models to estimate hazard ratios (HRs) and 95% CIs. We adjusted for noncompliance with screening in per-protocol analyses.^14^ All screened participants were included in per-protocol analyses, not just those screened by the main endoscopists. We used tests for interaction to examine heterogeneity of effect by detector ranking.

We calculated 3-year average rate ratios (RRs) with exact 95% CIs for distal CRC incidence and mortality for the first 16 years of follow-up, and estimated average annual RRs over years 6 to 10 and years 11 to 16. We calculated the NNS to prevent 1 CRC diagnosis or death, with 95% CIs, by dividing the number of screened participants by the total events prevented in those invited to screening.

We conducted analyses stratified by age (55–59, 60–64 y) and sex. For each stratum, we calculated ADRs, polyp detection rates, and average numbers of adenomas and polyps detected per 100 screened participants. We estimated HRs with 95% CIs to compare CRC incidence and mortality among the invited to screening and control arms, only presenting distal CRC outcomes.

We calculated an average ADR for all centers by methods comparable with those used in the BCSP; only including participants aged 55 years who did not have a repeat FS and not including adenomas detected at follow-up colonoscopy.

Analyses were performed using STATA/IC V.13.1 (Stata Statistical Software: release 13; StataCorp LP, College Station, TX). We set α at .05. The UKFSST is registered (ISRCTN28352761) and the protocol is available online.^15^ All authors had access to the data and reviewed and approved the final manuscript.

Ethics approval was obtained from local research ethics committees. All individuals offered FS screening provided written informed consent for the examination. Approval to obtain and process patient identifiable data without consent under Section 60 of the Health and Social Care Act 2001 was obtained from the Patient Information Advisory Group (reference: PIAG 4-07(j)_2002).

## Results

The mean age at randomization was 60 years, and 49% were men. The median follow-up time was 17.1 years (Table 1). Of 56,379 participants invited to screening, 40,085 (71%) attended. Of these, 38,550 (96%) were screened by 1 of the main trial endoscopists. Five, 4, and 4 centers were classified into the high-, intermediate-, and low-detector groups, respectively (Table 1).

**Table 1:** Participant Characteristics and Adenoma Detection Rates by Center Ranking

Center by main endoscopist ranking	Randomized	Men	Age at randomization, *y*	Follow-up time	Invited to screening	Screened	Screened by main endoscopist^a^	At least 1 adenoma^b^	At least 1 adenoma <10 mm^b^	At least 1 adenoma ≥10 mm^b^	At least 1 advanced adenoma^b^^,^^c^	Total adenomas, n	Average adenomas per 100 screened, n^b^	Referred for follow-up colonoscopy	Referred for colonoscopy surveillance
**Total**	**167,882**	**82,348 (49.1)**	**60.0 (2.9)**	**17.1 (16.4–17.7)**	**56,379**	**40,085 (71.1)**	**38,550 (96.2)**	**4609 (12.0)**	**3862 (10.0)**	**1005 (2.6)**	**1424 (3.7)**	**5856**	**15.2**	**1994 (5.2)**	**1624 (4.2)**
High detectors															
**Summary**	**63,334**	**31,094 (49.1)**	**60.0 (2.9)**	**17.1 (16.6–17.7)**	**21,222**	**15,212 (71.7)**	**14,633 (96.2)**	**2138 (14.6)**	**1801 (12.3)**	**461 (3.2)**	**625 (4.3)**	**2804**	**19.2**	**909 (6.2)**	**734 (5.0)**
1	12,732	6296 (49.5)	60.1 (2.9)	17.1 (17.1–18.0)	4260	3046 (71.5)	3007 (98.7)	466 (15.5)	370 (12.3)	133 (4.4)	178 (5.9)	638	21.2	263 (8.7)	219 (7.3)
2	12,939	6333 (48.9)	59.9 (2.9)	17.0 (16.6–17.5)	4342	3048 (70.2)	2644 (86.7)	388 (14.7)	338 (12.8)	75 (2.8)	115 (4.3)	505	19.1	162 (6.1)	140 (5.3)
3	12,489	6305 (50.5)	60.1 (2.8)	17.0 (17.0–17.4)	4193	3226 (76.9)	3175 (98.4)	462 (14.6)	399 (12.6)	93 (2.9)	106 (3.3)	604	19.0	145 (4.6)	119 (3.7)
4	12,148	5907 (48.6)	60.0 (2.9)	17.5 (17.2–17.7)	4066	2961 (72.8)	2904 (98.1)	417 (14.4)	353 (12.2)	86 (3.0)	112 (3.9)	550	18.9	178 (6.1)	147 (5.1)
5	13,026	6253 (48.0)	60.1 (2.8)	17.1 (16.4–17.7)	4361	2931 (67.2)	2903 (99.0)	405 (14.0)	341 (11.7)	74 (2.5)	114 (3.9)	507	17.5	161 (5.5)	109 (3.8)
Intermediate detectors															
**Summary**	**49,539**	**24,307 (49.1)**	**60.1 (2.9)**	**16.9 (16.2–17.6)**	**16,616**	**12,151 (73.1)**	**11,932 (98.2)**	**1379 (11.6)**	**1201 (10.1)**	**257 (2.2)**	**397 (3.3)**	**1743**	**14.6**	**540 (4.5)**	**461 (3.9)**
6	13,029	6312 (48.4)	60.0 (2.9)	17.0 (16.6–17.6)	4380	3184 (72.7)	3080 (96.7)	386 (12.5)	347 (11.3)	59 (1.9)	82 (2.7)	491	15.9	108 (3.5)	98 (3.2)
7	12,380	6195 (50.0)	60.1 (2.9)	17.1 (16.7–17.9)	4137	2997 (72.4)	2985 (99.6)	349 (11.7)	308 (10.3)	68 (2.3)	76 (2.5)	463	15.5	121 (4.1)	104 (3.5)
8	12,066	5938 (49.2)	60.2 (2.9)	16.7 (16.1–17.4)	4057	2939 (72.4)	2899 (98.6)	321 (11.1)	269 (9.3)	66 (2.3)	87 (3.0)	397	13.7	122 (4.2)	99 (3.4)
9	12,064	5862 (48.6)	60.0 (2.9)	16.6 (16.0–17.1)	4042	3031 (75.0)	2968 (97.9)	323 (10.9)	277 (9.3)	64 (2.2)	152 (5.1)	392	13.2	189 (6.4)	160 (5.4)
Low detectors															
**Summary**	**55,009**	**26,947 (49.0)**	**60.0 (2.9)**	**17.2 (16.4–18.0)**	**18,541**	**12,722 (68.6)**	**11,985 (94.2)**	**1092 (9.1)**	**860 (7.2)**	**287 (2.4)**	**402 (3.4)**	**1309**	**10.9**	**545 (4.5)**	**429 (3.6)**
10	12,246	6049 (49.4)	59.9 (2.9)	17.0 (16.4–17.8)	4120	2975 (72.2)	2948 (99.1)	282 (9.6)	203 (6.9)	92 (3.1)	120 (4.1)	334	11.3	171 (5.8)	140 (4.7)
11	12,480	6257 (50.1)	60.1 (2.8)	17.0 (16.3–17.4)	4191	2954 (70.5)	2478 (83.9)	235 (9.5)	201 (8.1)	44 (1.8)	79 (3.2)	286	11.5	97 (3.9)	89 (3.6)
12	16,417	7980 (48.6)	60.0 (2.9)	18.9 (17.5–18.9)	5553	3897 (70.2)	3893 (99.9)	349 (9.0)	279 (7.2)	89 (2.3)	133 (3.4)	416	10.7	179 (4.6)	127 (3.3)
13	13,866	6661 (48.0)	60.1 (2.8)	16.6 (14.1–17.4)	4677	2896 (61.9)	2666 (92.1)	226 (8.5)	177 (6.6)	62 (2.3)	70 (2.6)	273	10.2	98 (3.7)	73 (2.7)

NOTE. Data are n, n (%), mean (SD), or median (interquartile range), unless otherwise specified.

ADR, adenoma detection rate.

a A total of 1535 screened participants were excluded from our ADR calculations and were classed as not screened by 1 of the main endoscopists: 827 who were screened by an endoscopist other than 1 of the main endoscopists; 341 who had a colonoscopy rather than flexible sigmoidoscopy; and 367 who were screened during the first 2 months of screening at 1 center where the pathologist was found to be overdiagnosing adenomas.

b Only includes adenomas in participants screened by the main endoscopist at each center; percentages used the number screened by the main endoscopist as the denominator.

c Advanced adenomas were defined as adenomas ≥10 mm, with villous or tubulovillous histology, or high-grade dysplasia.

The average ADR for all centers was 12%, differing between 15%, 12%, and 9% in the high-, intermediate-, and low-detector groups, respectively. On average, 19, 15, and 11 adenomas were detected per 100 screened participants in each respective group (Table 1). Polyp detection rates and average numbers of polyps per 100 screened participants similarly decreased across the detector groups (Supplementary Table 1).

Detection rates of small adenomas were 12%, 10%, and 7%, respectively, in the high-, intermediate-, and low-detector groups. Corresponding figures for large adenomas were 3%, 2%, and 2%, and for advanced adenomas were 4%, 3%, and 3%, respectively (Table 1). Detection rates of small adenomas strongly correlated with the ADR (*P* < .001), whereas detection rates of large adenomas (*P* = .08) and advanced adenomas (*P* = .15) did not (Supplementary Table 2).

**Figure 1:**
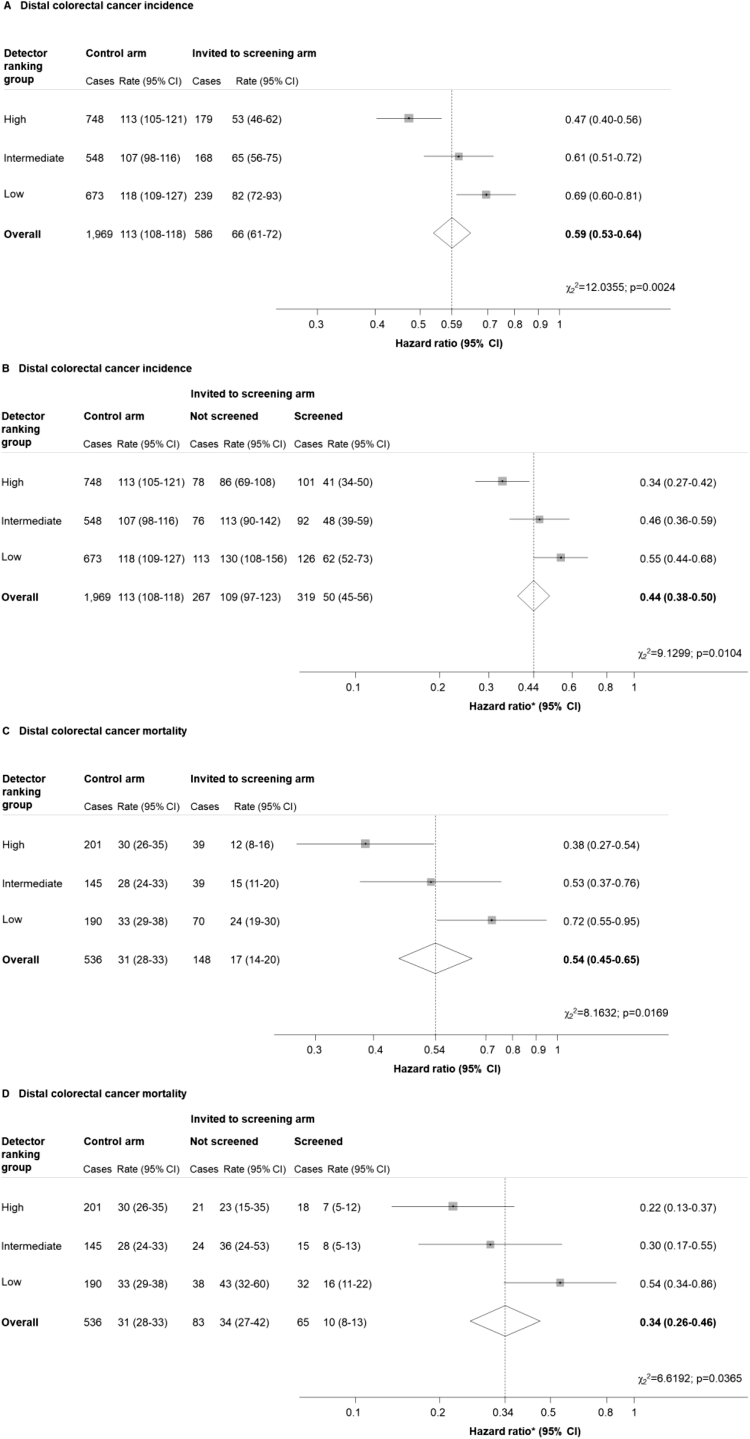
Hazard ratios for distal colorectal cancer incidence and mortality by detector ranking. Rates are per 100,000 person-years. (*A* and *C*) Hazard ratios are for the invited to screening vs control arm; (*B* and *D*) hazard ratios are for the screened vs control arm. *P* values from χ^2^ test for heterogeneity of effect by detector ranking. ∗Adjusted for noncompliance.

**Figure 2:**
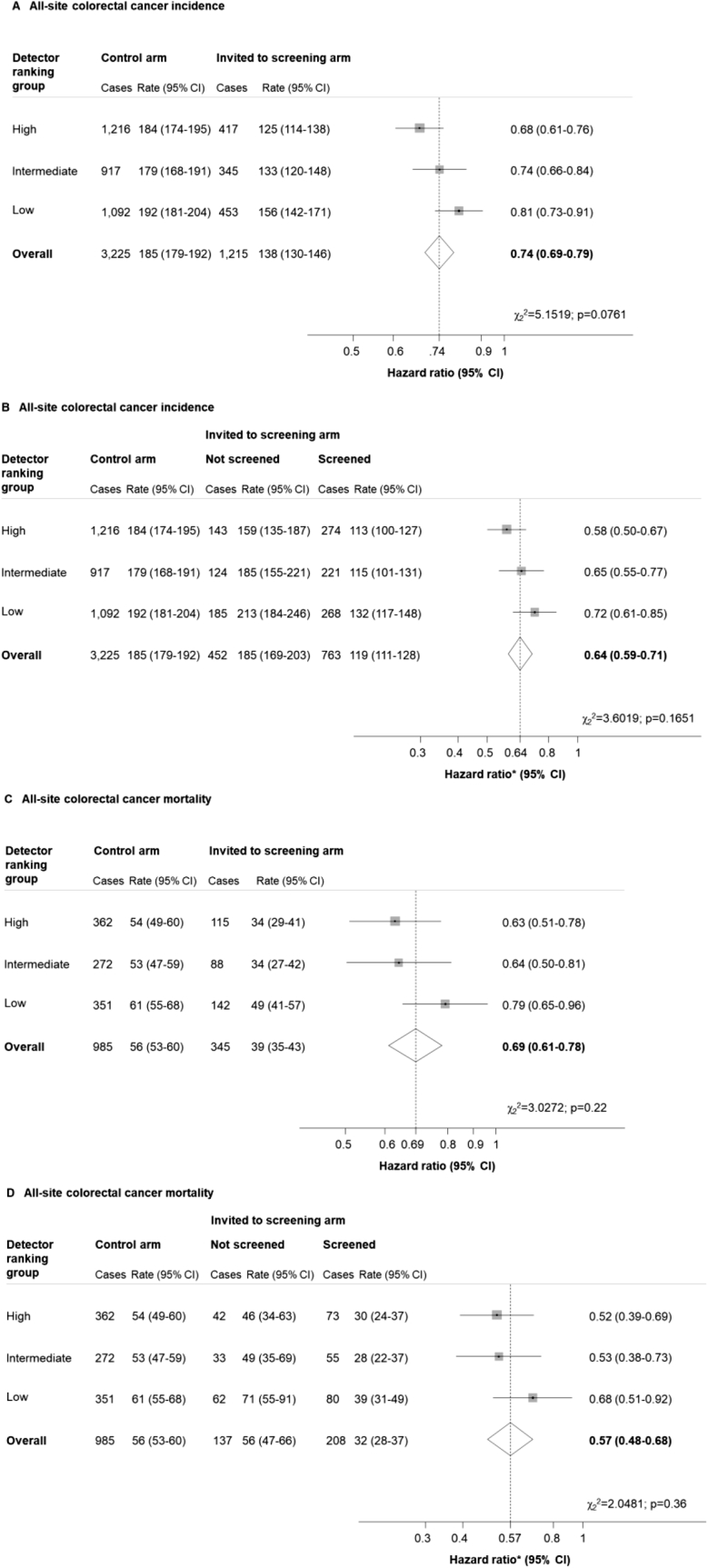
Hazard ratios for all-site colorectal cancer incidence and mortality by detector ranking. Rates are per 100,000 person-years. (*A* and *C*) Hazard ratios are for the invited to screening vs control arm; (*B* and *D*) hazard ratios are for the screened vs control arm. *P* values from χ^2^ test for heterogeneity of effect by detector ranking. ∗Adjusted for noncompliance.

Among those screened in the high-, intermediate-, and low-detector groups, 909 (6.2%), 540 (4.5%), and 545 (4.5%) were referred for colonoscopy, respectively (Table 1). Colonoscopy attendance was 95% or more in each group, and approximately half of colonoscopies were performed by the main trial endoscopists, although the proportion varied by center (data not shown). In the high-, intermediate-, and low-detector groups, 734 (5.0%), 461 (3.9%), and 429 (3.6%) screened participants were referred for surveillance, respectively (Table 1). During screening and follow-up, distal CRC was diagnosed in 927 (1.5%), 716 (1.4%), and 912 (1.7%) participants in the high-, intermediate-, and low-detector groups, respectively (Figure 1). All-site CRC was diagnosed in 1633 (2.6%), 1262 (2.5%), and 1545 (2.8%) participants in each respective group (Figure 2). Deaths resulting from distal CRCs occurred in 240 (0.4%), 184 (0.4%), and 260 (0.5%) participants in the high-, intermediate-, and low-detector groups, respectively (Figure 1). All-site CRC-related deaths occurred in 477 (0.8%), 360 (0.7%), and 493 (0.9%) participants in each respective group (Figure 2). Incidence and mortality rates of CRC showed little variation among controls (Figures 1 and 2).

### Distal Colorectal Cancer

Distal CRC incidence was reduced among those invited to screening compared with controls in all groups (high detectors: HR, 0.47; 95% CI, 0.40–0.56; intermediate detectors: HR, 0.61; 95% CI, 0.51–0.72; low detectors: HR, 0.69; 95% CI, 0.60–0.81) (Figure 1*A*), and even greater reductions were observed among those screened (high detectors: HR, 0.34; 95% CI, 0.27–0.42; intermediate detectors: HR, 0.46; 95% CI, 0.36–0.59; low detectors: HR, 0.55; 95% CI, 0.44–0.68) (Figure 1*B*).

Distal CRC mortality was reduced among those invited to screening in all groups (high detectors: HR, 0.38, 95% CI, 0.27–0.54; intermediate detectors: HR, 0.53; 95% CI, 0.37–0.76; low detectors: HR, 0.72; 95% CI, 0.55–0.95) (Figure 1*C*), and even more so among those screened (high detectors: HR, 0.22; 95% CI, 0.13–0.37; intermediate detectors: HR, 0.30; 95% CI, 0.17–0.55; low detectors: HR, 0.54; 95% CI, 0.34–0.86) (Figure 1*D*).

**Supplementary Figure 2:**
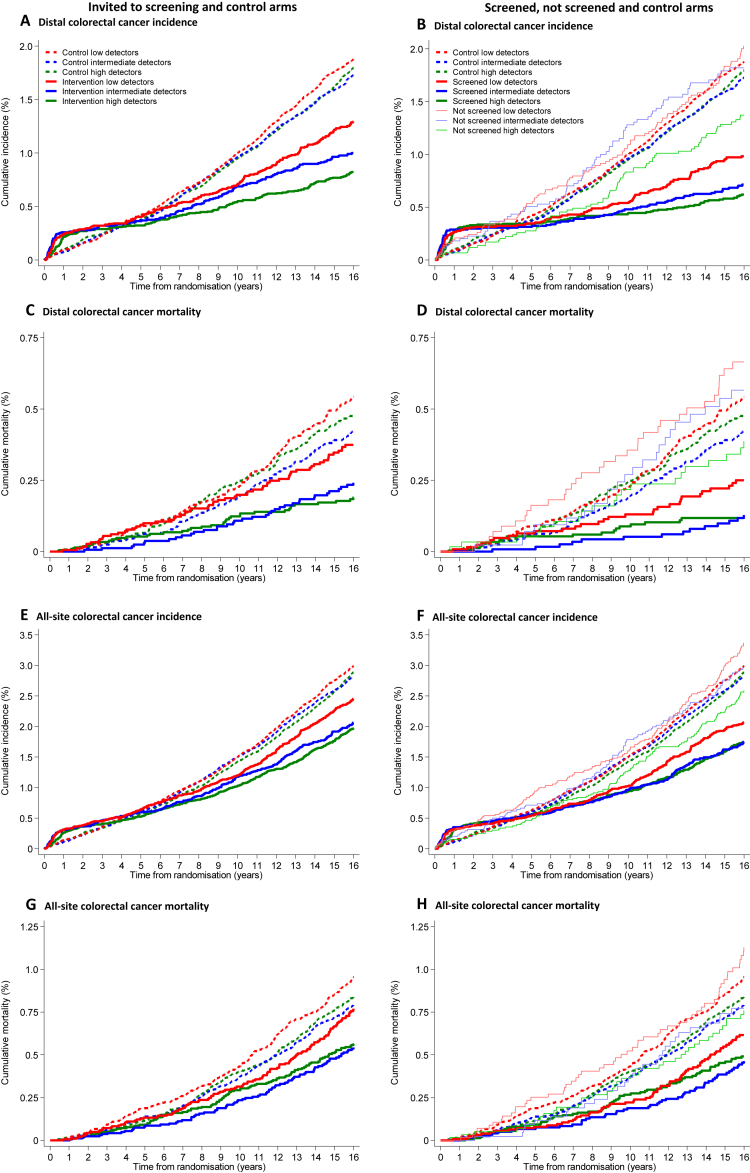
Cumulative colorectal cancer incidence and mortality by detector-ranking group. Kaplan–Meier estimates of cumulative colorectal cancer incidence and mortality by time from randomization and detector-ranking group. (*A*, *C*, *E*, and *G*) Intention-to-treat analyses, (*B*, *D*, *F*, and *H*) per-protocol analyses.

The effects of FS screening on distal CRC outcomes varied significantly by detector ranking (all *P* values < .05) (Figure 1, Supplementary Figure 2*A–D*).

### All-Site Colorectal Cancer

All-site CRC incidence was lower among those invited to screening compared with controls in all groups (high detectors: HR, 0.68; 95% CI, 0.61–0.76; intermediate detectors: HR, 0.74; 95% CI, 0.66–0.84; low detectors: HR, 0.81; 95% CI, 0.73–0.91) (Figure 2*A*), and even greater reductions were seen among those screened (high detectors: HR, 0.58; 95% CI, 0.50–0.67; intermediate detectors: HR, 0.65; 95% CI, 0.55–0.77; low detectors: HR, 0.72; 95% CI, 0.61–0.85) (Figure 2*B*).

All-site CRC mortality was reduced among those invited to screening in all groups (high detectors: HR, 0.63; 95% CI, 0.51–0.78; intermediate detectors: HR, 0.64; 95% CI, 0.50–0.81; low detectors: HR, 0.79; 95% CI, 0.65–0.96) (Figure 2*C*), and was reduced further among those screened (high detectors: HR, 0.52; 95% CI, 0.39–0.69; intermediate detectors: HR, 0.53; 95% CI, 0.38–0.73; low detectors: HR, 0.68; 95% CI, 0.51–0.92) (Figure 2*D*).

Although the effects of FS screening on all-site CRC outcomes varied by detector ranking, with the largest reductions seen in the high-detector group, the heterogeneity was not statistically significant (all *P* values ≥ .05) (Figure 2, Supplementary Figure 2*E–H*).

### Effects of Screening by Time Since Randomization

**Figure 3:**
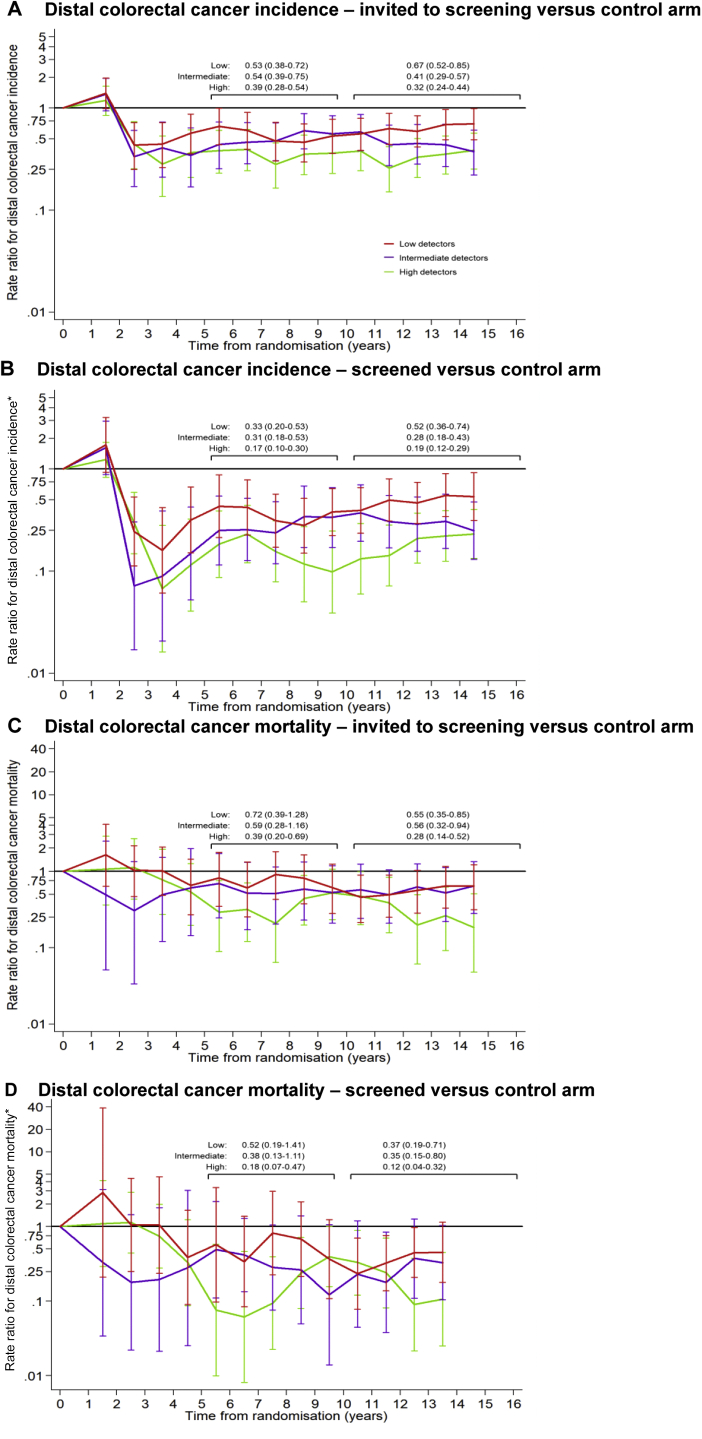
Average rate ratios for distal colorectal cancer incidence and mortality by detector ranking. Plotted points and 95% CIs are for the 3-year rolling average rate ratios. The average annual rate ratios over years 6 to 10 and years 11 to 16 are detailed. (A and C) Rate ratios for the invited to screening vs control arm; (B and D) rate ratios for the screened vs control arm. (*D*) The rate ratio is not presented for the final time point owing to few events in the high-detector group. ∗Adjusted for noncompliance.

Average RRs for distal CRC incidence peaked over the first 3 years of follow-up owing to the detection of prevalent CRCs at screening, but subsequently decreased to less than 1 in each detector group (Figure 3). In the high-, intermediate-, and low-detector groups, respectively, the per-protocol average annual RRs for distal CRC incidence were as follows: 0.17 (95% CI, 0.10–0.30), 0.31 (95% CI, 0.18–0.53), and 0.33 (95% CI, 0.20–0.53) over years 6 to 10, and 0.19 (95% CI, 0.12–0.29), 0.28 (95% CI, 0.18–0.43), and 0.52 (95% CI, 0.36–0.74) over years 11 to 16 (Figure 3*B*). For distal CRC mortality, corresponding figures were as follows: 0.18 (95% CI, 0.07–0.47), 0.38 (95% CI, 0.13–1.11), and 0.52 (95% CI, 0.19–1.41) over years 6 to 10, and 0.12 (95% CI, 0.04–0.32), 0.35 (95% CI, 0.15–0.80), and 0.37 (95% CI, 0.19–0.71) over years 11 to 16 (Figure 3*D*).

**Figure 4:**
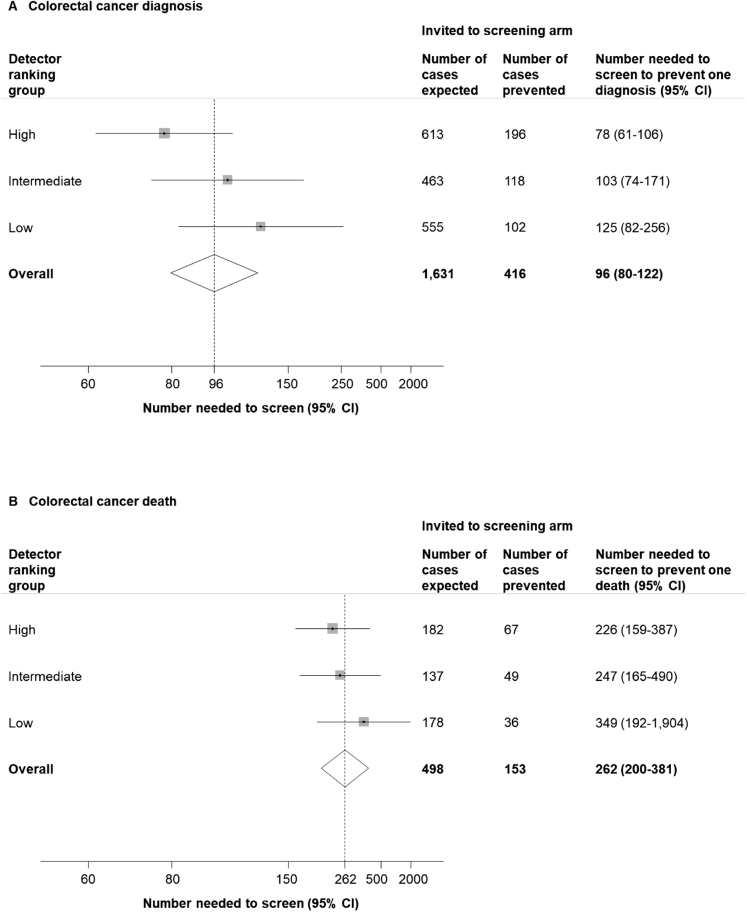
Number needed to screen to prevent 1 colorectal cancer (*A*) diagnosis or (*B*) death over 17 years by detector ranking.

The NNS to prevent 1 CRC diagnosis over 17 years was 78 (95% CI, 61–106), 103 (95% CI, 74–171), and 125 (95% CI, 82–256) in the high-, intermediate-, and low-detector groups, respectively. The NNS to prevent 1 CRC death followed the same pattern (Figure 4).

**Supplementary Figure 3:**
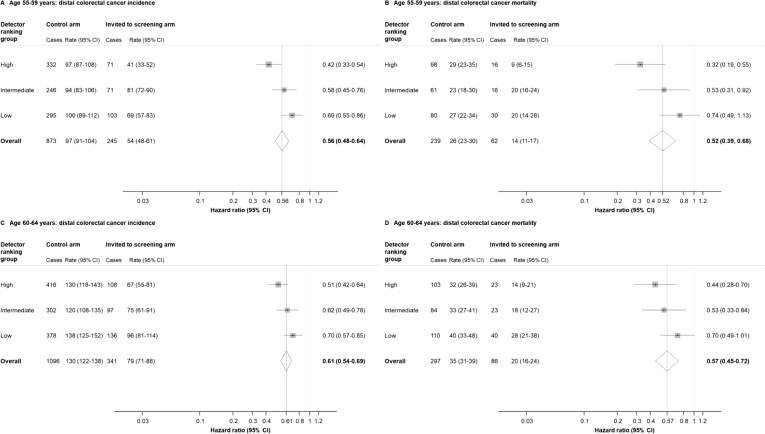
Hazard ratios for distal colorectal cancer incidence and mortality by detector*-*ranking group and age group in intention-to-treat analyses. Rates are per 100,000 person-years. Hazard ratios are for the invited to screening vs control arm in intention-to-treat analyses (*A* and *B*) among participants aged 55-59 years, (*C* and *D*) among participants aged 60-64 years.

**Supplementary Figure 4:**
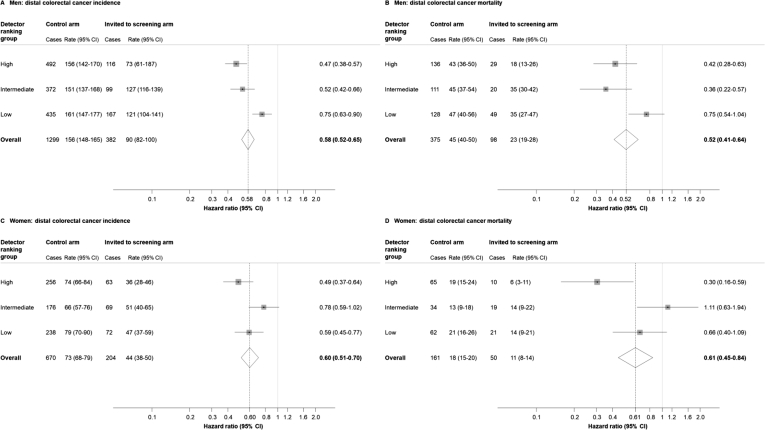
Hazard ratios for distal colorectal cancer incidence and mortality by detector*-*ranking group and sex in intention-to-treat analyses. Rates are per 100,000 person-years. Hazard ratios are for the invited to screening vs control arm in intention-to-treat analyses (*A* and *B*) among men, (*C* and *D*) among women.

In the high-, intermediate-, and low-detector groups, respectively, ADRs were 14%, 10%, and 9% among participants aged 55 to 59 years; 16%, 13%, and 9% among participants aged 60 to 64 years (Supplementary Table 3); 20%, 15%, and 12% among men; and 10%, 8%, and 6% among women (Supplementary Table 4). The HRs for distal CRC outcomes by age and sex followed the same general pattern by detector ranking as in the main analysis (Supplementary Figures 3 and 4).

The average ADR for all centers decreased from 12% (4605 of 38,550) to 9% (306 of 3376) when we only included participants aged 55 years without a repeat FS and only considered adenomas detected at FS (data not shown).

## Discussion

This study shows that a higher-quality screening endoscopy provides greater long-term protection against CRC. Examining 167,882 UKFSST participants, 40,085 of whom underwent FS screening, higher ADRs were associated with lower long-term CRC incidence and mortality after 17 years. Accordingly, the NNS to prevent 1 CRC diagnosis or death was lower for high-detector than intermediate-detector or low-detector ranking endoscopists.

Results were striking for distal CRC. Compared with participants screened by low-detectors, greater reductions in distal CRC incidence and mortality were seen among those screened by intermediate-detectors (54% vs 45% for incidence; 70% vs 46% for mortality), with even larger reductions among those screened by high-detectors (66% and 78% for incidence and mortality, respectively). The average annual RRs estimated reductions in distal CRC incidence of 81%, 72%, and 48% over years 11 to 16 among those screened by high-, intermediate-, and low-detectors, respectively.

Considering only 5% of participants were referred for follow-up colonoscopy and 4% were referred for surveillance, we conclude that the improved detection of adenomas at FS has a measurable impact on long-term distal CRC outcomes, even when there is infrequent colonoscopy use. It is possible that high-detectors also were more adept at polypectomy than intermediate- or low-detectors, and achieved more complete resection of detected lesions.

For all-site CRC, the effects of screening on incidence and mortality did not differ significantly by detector ranking. We previously showed that the UKFSST screening protocol had a negligible impact on proximal cancers, likely because few participants had their proximal colon examined by colonoscopy.^2^^,^^3^ Therefore, inclusion of proximal cancers in all-site calculations diluted the heterogeneity by detector ranking observed for distal outcomes.

In the UKFSST, small, large, and advanced adenomas all were detected more frequently in the high-detector than intermediate- or low-detector groups; however, only detection rates of small adenomas were correlated strongly with the ADR. This suggests that higher ADRs were driven by better detection of small adenomas, highlighting the significance of such adenomas, which, although have low malignant transformation rates,^16^ can develop into CRC over many years. However, the weaker correlation between the ADR and detection rates of large or advanced adenomas might be owing to the smaller numbers of these outcomes.

Several studies have associated higher ADRs with lower risks of postcolonoscopy CRC.^8^, ^9^, ^10^, ^11^ Results from 2 studies have indicated that postcolonoscopy CRC is several times more likely among individuals screened by endoscopists with ADRs less than 20% than among those screened by endoscopists with ADRs of 20% or greater.^10^^,^^11^ Another found that each 1% increase in ADR was associated with a 3% reduction in postcolonoscopy CRC risk.^8^ A similar association was seen between ADRs at screening FS and risk of distal CRC diagnosed within 4 years.^12^ However, these are short-term CRC outcomes, accounting for a minority of CRCs. Our unique data on the impact of ADRs on long-term CRC outcomes show that the ADR is a vitally important quality indicator, not just for FS screening but for colonoscopy screening and practice worldwide.

Numerous studies have considered how ADR improvement is achieved. In the German colonoscopy screening program, ADRs increased from 2003 to 2012.^17^ Possible reasons included increased training, higher-quality bowel preparation, and improvements in endoscopy equipment, which have led to higher ADRs in other studies.^10^^,^^18^ A study of FS screening in the BCSP found that insertion to the splenic flexure with a withdrawal time of 3.25 minutes or longer could help maximize ADRs.^19^

It is interesting to compare our ADRs with those observed in other settings. In 3 other FS screening trials, 7% to 16% of participants had distal adenomas detected during screening and follow-up colonoscopy.^20^ In the study of FS screening in the BCSP, the average ADR was 9%,^19^ matching our ADR when we used the age restriction and ADR definition applied in the BCSP. This provides external validation of our findings and indicates that the minimum recommended ADR of 6.8% for the BCSP needs to be reviewed.^21^

Potential drawbacks of pursuing higher ADRs include increased complication rates (eg, perforation, gastrointestinal bleeding).^6^ This was not seen in the UKFSST^7^ or the German screening program,^17^ but was observed in a study of screening colonoscopies in Austria, although the complication rate was less than 0.5% even in the highest ADR group.^22^ A focus on ADRs might result in more patients being diagnosed with multiple diminutive (≤5 mm) adenomas and recommended postpolypectomy surveillance, even though the CRC risk associated with these adenomas remains unclear.^23^

Limitations of our study included it being based on the ADRs of endoscopists who all were either gastroenterologists or surgeons. Screening can be performed by trained nurses and doctors, who may differ on other performance measures than the ADR compared with the trial endoscopists. The UKFSST only recruited individuals interested in screening; therefore, participants might have had a lower risk of colorectal neoplasia than the general population. However, we previously showed that CRC incidence among controls was similar to that in the general population.^2^

Strengths of this study included the large, high-quality data set; long follow-up period; and variation in ADRs. Participants were recruited from general practices around the UK that varied in size and setting. We obtained cancer and death data from national data sets, which provide a high level of population coverage and enabled us to follow up participants even if they migrated within the UK; the loss to follow-up rate therefore was low. Rates of outcomes showed little variation among controls, indicating that the results were not confounded by baseline differences in CRC risk.

## Conclusions

Higher ADRs at screening FS provide greater long-term protection against CRC incidence and mortality. In our analysis, distal CRC incidence and mortality were reduced by 66% and 78%, respectively, among individuals screened by high-detectors. This effect is substantially larger than has been observed to date. We found that higher ADRs reflected improved detection of small adenomas. These findings are vitally important to CRC screening programs involving either FS or colonoscopy. They highlight the need for quality assurance and careful monitoring of ADRs to realize the full public health benefits of endoscopic screening.

## Figures and Tables

**Supplementary Table 1:** Polyp Detection Rates by Center Ranking

Center by main endoscopist ranking	Screened by main endoscopist		At least 1 polyp^a^		At least 1 polyp <10 mm^a^		At least 1 polyp ≥10 mm^a^		Total polyps, n^a^	Average polyps per 100 screened, n^a^
**Total**	**38,550**	**(96.2)**	**9698**	**(25.2)**	**9104**	**(23.6)**	**1122**	**(2.9)**	**18,464**	**47.9**
High detectors										
**Summary**	**14,633**	**(96.2)**	**4611**	**(31.5)**	**4354**	**(29.8)**	**518**	**(3.5)**	**10,068**	**68.8**
1	3007	(98.7)	1211	(40.3)	1143	(38.0)	150	(5.0)	3399	113.0
2	2644	(86.7)	807	(30.5)	769	(29.1)	86	(3.3)	1765	66.8
3	3175	(98.4)	945	(29.8)	898	(28.3)	99	(3.1)	1840	58.0
4	2904	(98.1)	805	(27.7)	755	(26.0)	100	(3.4)	1455	50.1
5	2903	(99.0)	843	(29.0)	789	(27.2)	83	(2.9)	1609	55.4
Intermediate detectors										
**Summary**	**11,932**	**(98.2)**	**2914**	**(24.4)**	**2774**	**(23.2)**	**290**	**(2.4)**	**5202**	**43.6**
6	3080	(96.7)	751	(24.4)	719	(23.3)	63	(2.0)	1422	46.2
7	2985	(99.6)	821	(27.5)	798	(26.7)	87	(2.9)	1707	57.2
8	2899	(98.6)	623	(21.5)	574	(19.8)	68	(2.3)	904	31.2
9	2968	(97.9)	719	(24.2)	683	(23.0)	72	(2.4)	1169	39.4
Low detectors										
**Summary**	**11,985**	**(94.2)**	**2173**	**(18.1)**	**1976**	**(16.5)**	**314**	**(2.6)**	**3194**	**26.6**
10	2948	(99.1)	568	(19.3)	503	(17.1)	104	(3.5)	833	28.3
11	2478	(83.9)	401	(16.2)	372	(15.0)	49	(2.0)	591	23.8
12	3893	(99.9)	816	(21.0)	757	(19.4)	92	(2.4)	1230	31.6
13	2666	(92.1)	388	(14.6)	344	(12.9)	69	(2.6)	540	20.3

NOTE. Data are n, n (%), or mean.

a Only includes polyps detected in people screened by the main endoscopist at each center; percentages are calculated using the number screened by the main endoscopist as the denominator.

**Supplementary Table 2:** Correlation Between the ADR and Detection Rates of Small (<10 mm), Large (≥10 mm), and Advanced Lesions

Detection rate	Correlation with ADR^a^	
	Rho	*P* value
Adenomas, <10 mm	0.96	<.001
Adenomas, ≥10 mm	0.51	.08
Advanced adenomas^b^	0.42	.15
Polyps	0.97	<.001
Polyps, <10 mm	0.95	<.001
Polyps, ≥10 mm	0.57	.04

ADR, adenoma detection rate.

a Spearman rank correlation coefficient (Rho) and associated *P* value was used to assess the correlation between the ADR and detection rates of small (<10 mm) adenomas/polyps, large (≥10 mm) adenomas/polyps, and advanced adenomas.

b Advanced adenomas were defined as adenomas ≥10 mm, with villous or tubulovillous histology, or high-grade dysplasia.

**Supplementary Table 3:** Adenoma Detection Rates by Center Ranking and Age Group

Age, *y*	Center by main endoscopist ranking	Invited to screening	Screened		Screened by main endoscopist		At least 1 adenoma^a^		At least 1 adenoma <10 mm^a^		At least 1 adenoma ≥10 mm^a^		At least 1 advanced adenoma^a^^,^^b^		Total adenomas, n^a^	Average adenomas per 100 screened, n^a^	Referred for follow-up colonoscopy		Referred for colonoscopy surveillance	
**55–59**	**Total**	**28,248**	**20,202**	**(71.5)**	**19,404**	**(96.0)**	**2163**	**(11.1)**	**1827**	**(9.4)**	**435**	**(2.2)**	**643**	**(3.3)**	**2710**	**14.0**	**893**	**(4.6)**	**748**	**(3.9)**
	High detectors																			
	**Summary**	**10,713**	**7724**	**(72.1)**	**7424**	**(96.1)**	**1017**	**(13.7)**	**861**	**(11.6)**	**205**	**(2.8)**	**290**	**(3.9)**	**1309**	**17.6**	**417**	**(5.6)**	**345**	**(4.6)**
	1	2104	1498	(71.2)	1476	(98.5)	217	(14.7)	175	(11.9)	58	(3.9)	82	(5.6)	290	19.6	117	(7.9)	100	(6.8)
	2	2269	1591	(70.1)	1372	(86.2)	191	(13.9)	165	(12.0)	36	(2.6)	60	(4.4)	243	17.7	89	(6.5)	77	(5.6)
	3	2115	1626	(76.9)	1605	(98.7)	236	(14.7)	209	(13.0)	42	(2.6)	50	(3.1)	292	18.2	62	(3.9)	53	(3.3)
	4	2070	1532	(74.0)	1510	(98.6)	199	(13.2)	166	(11.0)	39	(2.6)	49	(3.2)	267	17.7	84	(5.6)	71	(4.7)
	5	2155	1477	(68.5)	1461	(98.9)	174	(11.9)	146	(10.0)	30	(2.1)	49	(3.4)	217	14.9	65	(4.4)	44	(3.0)
	Intermediate detectors																			
	**Summary**	**8190**	**6000**	**(73.3)**	**5883**	**(98.1)**	**602**	**(10.2)**	**531**	**(9.0)**	**95**	**(1.6)**	**156**	**(2.7)**	**753**	**12.8**	**218**	**(3.7)**	**192**	**(3.3)**
	6	2149	1560	(72.6)	1512	(96.9)	164	(10.8)	142	(9.4)	27	(1.8)	33	(2.2)	201	13.3	47	(3.1)	42	(2.8)
	7	2023	1493	(73.8)	1487	(99.6)	148	(10.0)	130	(8.7)	26	(1.7)	30	(2.0)	205	13.8	51	(3.4)	47	(3.2)
	8	1981	1438	(72.6)	1413	(98.3)	163	(11.5)	147	(10.4)	20	(1.4)	32	(2.3)	192	13.6	47	(3.3)	42	(3.0)
	9	2037	1509	(74.1)	1471	(97.5)	127	(8.6)	112	(7.6)	22	(1.5)	61	(4.1)	155	10.5	73	(5.0)	61	(4.1)
	Low detectors																			
	**Summary**	**9345**	**6478**	**(69.3)**	**6097**	**(94.1)**	**544**	**(8.9)**	**435**	**(7.1)**	**135**	**(2.2)**	**197**	**(3.2)**	**648**	**10.6**	**258**	**(4.2)**	**211**	**(3.5)**
	10	2108	1512	(71.7)	1501	(99.3)	152	(10.1)	114	(7.6)	44	(2.9)	62	(4.1)	183	12.2	83	(5.5)	66	(4.4)
	11	2049	1474	(71.9)	1224	(83.0)	113	(9.2)	93	(7.6)	26	(2.1)	42	(3.4)	142	11.6	47	(3.8)	45	(3.7)
	12	2824	2009	(71.1)	2006	(99.9)	167	(8.3)	136	(6.8)	39	(1.9)	62	(3.1)	195	9.7	81	(4.0)	65	(3.2)
	13	2364	1483	(62.7)	1366	(92.1)	112	(8.2)	92	(6.7)	26	(1.9)	31	(2.3)	128	9.4	47	(3.4)	35	(2.6)
**60–64**	**Total**	**28,131**	**19,883**	**(70.7)**	**19,146**	**(96.3)**	**2446**	**(12.8)**	**2035**	**(10.6)**	**570**	**(3.0)**	**781**	**(4.1)**	**3146**	**16.4**	**1101**	**(5.8)**	**876**	**(4.6)**
	High detectors																			
	**Summary**	**10,509**	**7488**	**(71.3)**	**7209**	**(96.3)**	**1121**	**(15.6)**	**940**	**(13.0)**	**256**	**(3.6)**	**335**	**(4.6)**	**1495**	**20.7**	**492**	**(6.8)**	**389**	**(5.4)**
	1	2156	1548	(71.8)	1531	(98.9)	249	(16.3)	195	(12.7)	75	(4.9)	96	(6.3)	348	22.7	146	(9.5)	119	(7.8)
	2	2073	1457	(70.3)	1272	(87.3)	197	(15.5)	173	(13.6)	39	(3.1)	55	(4.3)	262	20.6	73	(5.7)	63	(5.0)
	3	2078	1600	(77.0)	1570	(98.1)	226	(14.4)	190	(12.1)	51	(3.2)	56	(3.6)	312	19.9	83	(5.3)	66	(4.2)
	4	1996	1429	(71.6)	1394	(97.6)	218	(15.6)	187	(13.4)	47	(3.4)	63	(4.5)	283	20.3	94	(6.7)	76	(5.5)
	5	2206	1454	(65.9)	1442	(99.2)	231	(16.0)	195	(13.5)	44	(3.1)	65	(4.5)	290	20.1	96	(6.7)	65	(4.5)
	Intermediate detectors																			
	**Summary**	**8426**	**6151**	**(73.0)**	**6049**	**(98.3)**	**777**	**(12.8)**	**670**	**(11.1)**	**162**	**(2.7)**	**241**	**(4.0)**	**990**	**16.4**	**322**	**(5.3)**	**269**	**(4.4)**
	6	2231	1624	(72.8)	1568	(96.6)	222	(14.2)	205	(13.1)	32	(2.0)	49	(3.1)	290	18.5	61	(3.9)	56	(3.6)
	7	2114	1504	(71.1)	1498	(99.6)	201	(13.4)	178	(11.9)	42	(2.8)	46	(3.1)	258	17.2	70	(4.7)	57	(3.8)
	8	2076	1501	(72.3)	1486	(99.0)	158	(10.6)	122	(8.2)	46	(3.1)	55	(3.7)	205	13.8	75	(5.0)	57	(3.8)
	9	2005	1522	(75.9)	1497	(98.4)	196	(13.1)	165	(11.0)	42	(2.8)	91	(6.1)	237	15.8	116	(7.7)	99	(6.6)
	Low detectors																			
	**Summary**	**9196**	**6244**	**(67.9)**	**5888**	**(94.3)**	**548**	**(9.3)**	**425**	**(7.2)**	**152**	**(2.6)**	**205**	**(3.5)**	**661**	**11.2**	**287**	**(4.9)**	**218**	**(3.7)**
	10	2012	1463	(72.7)	1447	(98.9)	130	(9.0)	89	(6.2)	48	(3.3)	58	(4.0)	151	10.4	88	(6.1)	74	(5.1)
	11	2142	1480	(69.1)	1254	(84.7)	122	(9.7)	108	(8.6)	18	(1.4)	37	(3.0)	144	11.5	50	(4.0)	44	(3.5)
	12	2729	1888	(69.2)	1887	(99.9)	182	(9.6)	143	(7.6)	50	(2.6)	71	(3.8)	221	11.7	98	(5.2)	62	(3.3)
	13	2313	1413	(61.1)	1300	(92.0)	114	(8.8)	85	(6.5)	36	(2.8)	39	(3.0)	145	11.2	51	(3.9)	38	(2.9)

NOTE. Data are shown as n, n (%), or mean.

a Only includes adenomas detected in people screened by the main endoscopist in each center; percentages are calculated using the number screened by the main endoscopist as the denominator.

b Advanced adenomas were defined as adenomas ≥10 mm, with villous or tubulovillous histology, or high-grade dysplasia.

**Supplementary Table 4:** Adenoma Detection Rates by Center Ranking and Sex

Sex	Center by main endoscopist ranking	Invited to screening	Screened		Screened by main endoscopist		At least 1 adenoma^a^		At least 1 adenoma <10 mm^a^		At least 1 adenoma ≥10 mm^a^		At least 1 advanced adenoma^a^^,^^b^		Total adenomas, n^a^	Average adenomas per 100 screened, n^a^	Referred for follow-up colonoscopy		Referred for colonoscopy surveillance	
Men	**Total**	**27,665**	**20,248**	**(73.2)**	**19,487**	**(96.2)**	**3090**	**(15.9)**	**2582**	**(13.2)**	**715**	**(3.7)**	**982**	**(5.0)**	**4099**	**21.0**	**1366**	**(7.0)**	**1118**	**(5.7)**
	High detectors																			
	**Summary**	**10,415**	**7670**	**(73.6)**	**7371**	**(96.1)**	**1437**	**(19.5)**	**1218**	**(16.5)**	**323**	**(4.4)**	**425**	**(5.8)**	**1983**	**26.9**	**609**	**(8.3)**	**495**	**(6.7)**
	1	2108	1547	(73.4)	1528	(98.8)	313	(20.5)	249	(16.3)	92	(6.0)	120	(7.9)	447	29.3	175	(11.5)	148	(9.7)
	2	2123	1547	(72.9)	1326	(85.7)	260	(19.6)	223	(16.8)	60	(4.5)	80	(6.0)	360	27.1	101	(7.6)	89	(6.7)
	3	2116	1654	(78.2)	1628	(98.4)	307	(18.9)	267	(16.4)	67	(4.1)	73	(4.5)	426	26.2	100	(6.1)	82	(5.0)
	4	1976	1472	(74.5)	1457	(99.0)	278	(19.1)	234	(16.1)	62	(4.3)	78	(5.4)	384	26.4	126	(8.6)	105	(7.2)
	5	2092	1450	(69.3)	1432	(98.8)	279	(19.5)	245	(17.1)	42	(2.9)	74	(5.2)	366	25.6	107	(7.5)	71	(5.0)
	Intermediate detectors																			
	**Summary**	**8157**	**6146**	**(75.3)**	**6040**	**(98.3)**	**909**	**(15.0)**	**795**	**(13.2)**	**176**	**(2.9)**	**258**	**(4.3)**	**1197**	**19.8**	**360**	**(6.0)**	**305**	**(5.0)**
	6	2118	1597	(75.4)	1553	(97.2)	264	(17.0)	235	(15.1)	45	(2.9)	60	(3.9)	349	22.5	78	(5.0)	71	(4.6)
	7	2074	1548	(74.6)	1544	(99.7)	240	(15.5)	216	(14.0)	42	(2.7)	46	(3.0)	325	21.0	79	(5.1)	65	(4.2)
	8	1999	1468	(73.4)	1443	(98.3)	209	(14.5)	177	(12.3)	46	(3.2)	60	(4.2)	275	19.1	87	(6.0)	70	(4.9)
	9	1966	1533	(78.0)	1500	(97.8)	196	(13.1)	167	(11.1)	43	(2.9)	92	(6.1)	248	16.5	116	(7.7)	99	(6.6)
	Low detectors																			
	**Summary**	**9093**	**6432**	**(70.7)**	**6076**	**(94.5)**	**744**	**(12.2)**	**569**	**(9.4)**	**216**	**(3.6)**	**299**	**(4.9)**	**919**	**15.1**	**397**	**(6.5)**	**318**	**(5.2)**
	10	2040	1516	(74.3)	1506	(99.3)	198	(13.1)	139	(9.2)	68	(4.5)	89	(5.9)	239	15.9	122	(8.1)	103	(6.8)
	11	2091	1521	(72.7)	1288	(84.7)	175	(13.6)	150	(11.6)	34	(2.6)	61	(4.7)	220	17.1	73	(5.7)	65	(5.0)
	12	2705	1963	(72.6)	1961	(99.9)	230	(11.7)	171	(8.7)	73	(3.7)	103	(5.3)	280	14.3	137	(7.0)	102	(5.2)
	13	2257	1432	(63.4)	1321	(92.2)	141	(10.7)	109	(8.3)	41	(3.1)	46	(3.5)	180	13.6	65	(4.9)	48	(3.6)
Women	**Total**	**28,714**	**19,837**	**(69.1)**	**19,063**	**(96.1)**	**1519**	**(8.0)**	**1280**	**(6.7)**	**290**	**(1.5)**	**442**	**(2.3)**	**1757**	**9.2**	**628**	**(3.3)**	**506**	**(2.7)**
	High detectors																			
	**Summary**	**10,807**	**7542**	**(69.8)**	**7262**	**(96.3)**	**701**	**(9.7)**	**583**	**(8.0)**	**138**	**(1.9)**	**200**	**(2.8)**	**821**	**11.3**	**300**	**(4.1)**	**239**	**(3.3)**
	1	2152	1499	(69.7)	1479	(98.7)	153	(10.3)	121	(8.2)	41	(2.8)	58	(3.9)	191	12.9	88	(5.9)	71	(4.8)
	2	2219	1501	(67.6)	1318	(87.8)	128	(9.7)	115	(8.7)	15	(1.1)	35	(2.7)	145	11.0	61	(4.6)	51	(3.9)
	3	2077	1572	(75.7)	1547	(98.4)	155	(10.0)	132	(8.5)	26	(1.7)	33	(2.1)	178	11.5	45	(2.9)	37	(2.4)
	4	2090	1489	(71.2)	1447	(97.2)	139	(9.6)	119	(8.2)	24	(1.7)	34	(2.3)	166	11.5	52	(3.6)	42	(2.9)
	5	2269	1481	(65.3)	1471	(99.3)	126	(8.6)	96	(6.5)	32	(2.2)	40	(2.7)	141	9.6	54	(3.7)	38	(2.6)
	Intermediate detectors																			
	**Summary**	**8459**	**6005**	**(71.0)**	**5892**	**(98.1)**	**470**	**(8.0)**	**406**	**(6.9)**	**81**	**(1.4)**	**139**	**(2.4)**	**546**	**9.3**	**180**	**(3.1)**	**156**	**(2.6)**
	6	2262	1587	(70.2)	1527	(96.2)	122	(8.0)	112	(7.3)	14	(0.9)	22	(1.4)	142	9.3	30	(2.0)	27	(1.8)
	7	2063	1449	(70.2)	1441	(99.4)	109	(7.6)	92	(6.4)	26	(1.8)	30	(2.1)	138	9.6	42	(2.9)	39	(2.7)
	8	2058	1471	(71.5)	1456	(99.0)	112	(7.7)	92	(6.3)	20	(1.4)	27	(1.9)	122	8.4	35	(2.4)	29	(2.0)
	9	2076	1498	(72.2)	1468	(98.0)	127	(8.7)	110	(7.5)	21	(1.4)	60	(4.1)	144	9.8	73	(5.0)	61	(4.2)
	Low detectors																			
	**Summary**	**9448**	**6290**	**(66.6)**	**5909**	**(93.9)**	**348**	**(5.9)**	**291**	**(4.9)**	**71**	**(1.2)**	**103**	**(1.7)**	**390**	**6.6**	**148**	**(2.5)**	**111**	**(1.9)**
	10	2080	1459	(70.1)	1442	(98.8)	84	(5.8)	64	(4.4)	24	(1.7)	31	(2.1)	95	6.6	49	(3.4)	37	(2.6)
	11	2100	1433	(68.2)	1190	(83.0)	60	(5.0)	51	(4.3)	10	(0.8)	18	(1.5)	66	5.5	24	(2.0)	24	(2.0)
	12	2848	1934	(67.9)	1932	(99.9)	119	(6.2)	108	(5.6)	16	(0.8)	30	(1.6)	136	7.0	42	(2.2)	25	(1.3)
	13	2420	1464	(60.5)	1345	(91.9)	85	(6.3)	68	(5.1)	21	(1.6)	24	(1.8)	93	6.9	33	(2.5)	25	(1.9)

NOTE. Data are shown as n, n (%), or mean.

a Only includes adenomas detected in people screened by the main endoscopist at each center; percentages are calculated using the number screened by the main endoscopist as the denominator.

b Advanced adenomas were defined as adenomas ≥10 mm, with villous or tubulovillous histology, or high-grade dysplasia.
